# TMD splitting functions in $k_T$ factorization: the real contribution to the gluon-to-gluon splitting

**DOI:** 10.1140/epjc/s10052-018-5634-2

**Published:** 2018-03-02

**Authors:** M. Hentschinski, A. Kusina, K. Kutak, M. Serino

**Affiliations:** 1 Departamento de Actuaria, Física y Matemáticas, Universidad de las Americas Puebla, Santa Catarina Martir, 72820 Puebla, Mexico; 2 The H. Niewodniczański Institute of Nuclear Physics, Polish Academy of Sciences, ul. Radzikowskiego 152, 31-342 Cracow, Poland; 3 Department of Physics, Ben Gurion University of the Negev, 8410501 Beer Sheva, Israel

## Abstract

We calculate the transverse momentum dependent gluon-to-gluon splitting function within $k_T$-factorization, generalizing the framework employed in the calculation of the quark splitting functions in Hautmann et al. (Nucl Phys B 865:54-66, arXiv:1205.1759, 2012), Gituliar et al. (JHEP 01:181, arXiv:1511.08439, 2016), Hentschinski et al. (Phys Rev D 94(11):114013, arXiv:1607.01507, 2016) and demonstrate at the same time the consistency of the extended formalism with previous results. While existing versions of $k_T$ factorized evolution equations contain already a gluon-to-gluon splitting function *i.e.* the leading order Balitsky–Fadin–Kuraev–Lipatov (BFKL) kernel or the Ciafaloni–Catani–Fiorani–Marchesini (CCFM) kernel, the obtained splitting function has the important property that it reduces both to the leading order BFKL kernel in the high energy limit, to the Dokshitzer–Gribov–Lipatov–Altarelli–Parisi (DGLAP) gluon-to-gluon splitting function in the collinear limit as well as to the CCFM kernel in the soft limit. At the same time we demonstrate that this splitting kernel can be obtained from a direct calculation of the QCD Feynman diagrams, based on a combined implementation of the Curci-Furmanski-Petronzio formalism for the calculation of the collinear splitting functions and the framework of high energy factorization.

## Introduction

Parton distributions functions (PDFs) are crucial elements of collider phenomenology. In presence of a hard scale *M* with $M \gg \Lambda _{\text {QCD}}$ and $\Lambda _{\text {QCD}}$ the QCD characteristic scale of the order of a few hundred MeV, factorization theorems allow to express cross-sections as convolutions of parton densities and hard matrix elements, where the latter are calculated within perturbative QCD [4]. This was first achieved within the framework of collinear factorization [5–7], where the incoming partons are taken to be collinear with the respective mother hadron. Calculating hard matrix elements to higher orders in the strong coupling constant, one can systematically improve the precision of the theoretical prediction, by incorporating more loops and more emissions of real partons. These extra emissions allow to improve the kinematic approximation inherent to the leading order description.

As an alternative to improving the kinematic description through the calculation of higher order corrections, one may attempt to treat kinematics exactly from the very beginning, *i.e.* to account for the bulk of kinematic effects already at leading order. An important example of such kinematic effects is the transverse momentum $k_T$ of the initial state partons, which is set to zero within collinear factorization. Therefore, in collinear factorization, these effects can only be recovered through the calculation of higher order corrections, in particular through real parton emissions from initial state particles, which generate finite transverse momenta for the initial state partons of the observed hard event. Schemes which provide an improved kinematic description already at the leading order involve in general Transverse-Momentum-Dependent (TMD) or ‘unintegrated’ PDFs.^1^

TMD PDFs arise naturally in regions of phase space characterized by a hierarchy of scales. A particularly interesting example is provided by the so called low *x* region, where *x* is the ratio of the hard scale $M^2$ of the process and the center-of-mass energy squared *s*. The low *x* region corresponds therefore to the hierarchy $s \gg M^2 \gg \Lambda _{\text {QCD}}^2$. In such a kinematical setup, large logarithms $\ln 1/x$ can compensate for the smallness of the perturbative strong coupling $\alpha _s$ and it is necessary to resum terms $\left( \alpha _s \ln 1/x \right) ^n$ to all orders to maintain the predictive power of the perturbative expansion. Such a resummation is achieved by the Balitsky-Fadin-Kuraev-Lipatov (BFKL) [9–12] evolution equation. Its formulation is based on factorization of QCD amplitudes in the high energy limit, $s \gg M^2$. As a natural by-product of such a factorization, one obtains QCD cross-sections as convolutions in transverse momentum: in particular, cross-sections are automatically factorized into $k_T$ dependent impact factors and the BFKL Green’s function. Matching of high energy factorization to collinear factorization which identifies properly normalized impact factors and Green’s function with unintegrated gluon density and $k_T$-dependent perturbative coefficients is then achieved by so-called $k_T$-factorization [13]; see [14–17] for studies of various processes within this scheme.

While high energy factorization provides a well defined framework for calculations of evolution kernels and coefficient functions also beyond leading order, the applicability of the results is naturally limited to the low *x* limit of hard scattering events. If the ensuing formalism is naïvely extrapolated to intermediate or even large values of *x*, the framework is naturally confronted with a series of problems and short-comings. To name a few, contributions of quarks to the evolution arise as a pure next-to-leading order (NLO) effect and elementary vertices violate energy conservation *i.e.* conservation of the hadron longitudinal momentum fraction. While these effects are subleading in the strict limit $x \rightarrow 0$, they become sizeable if intermediate values of *x* are reached. The only way to account for such effects within the high energy factorization framework is through the determination of perturbative higher order terms. Apart from the direct calculation of perturbative higher order corrections [18, 19], this can be achieved through including a resummation of terms, which restore subleading but numerically relevant pieces of the Dokshitzer–Gribov–Lipatov–Altarelli–Parisi (DGLAP) [20–22] splitting functions [23–28]; for early attempts to unify the DGLAP and BFKL approaches see [25, 29] as well as the more recent attempt [30]. Even though these resummations have been successful in stabilizing low *x* evolution into the region of intermediate $x \sim 10^{-2}$, extrapolations to larger values of *x* are still prohibited. Moreover, by merely resumming and calculating higher order corrections within the BFKL formalism, one essentially repeats the program initially outlined for collinear factorization: higher order corrections are calculated to account for kinematic effects which are beyond the regarding factorization scheme.

**Fig. 1:**
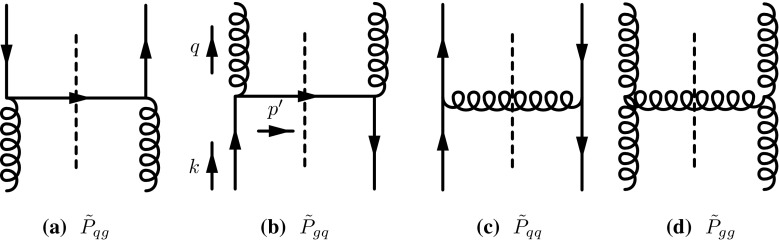
Squared matrix elements for the determination of the real contributions to the splitting functions à-la Curci-Furmanski-Petronzio. Lower (incoming) lines carry always momentum *k*, upper (outgoing) lines carry momentum *q*

To arrive at a framework which avoids the need to account for kinematic effects through the calculation of higher order corrections, it is therefore necessary to arrive at a scheme which accounts for both DGLAP (conservation of the longitudinal momentum fraction) and BFKL (conservation of the transverse momentum) kinematics. Note that the mere definition of such a scheme is difficult: neither the hard scale of the process (as in the case of DGLAP evolution) nor *x* (BFKL evolution) provides at first a suitable expansion parameter, if one desires to keep exact kinematics in both variables. To overcome these difficulties, we follow here a proposal initially outlined in [31]: There, following the all-order definition of DGLAP splitting function within the Curci–Furmanski–Petronzio (CFP) [32] formalism, the low *x* resummed DGLAP splitting functions have been constructed. As an ingredient for such a resummation program, the authors of [31] were able to define a TMD gluon-to-quark splitting function ${\tilde{P}}_{qg}$, both exact in transverse momentum and longitudinal momentum fraction (hereafter, we will use the symbol ${\tilde{P}}$ to indicate a transverse momentum dependent splitting function). While the TMD gluon-to-quark splitting function is well defined within the low *x* resummation program of [31], the same is not true for the other splitting kernels. In [1] it was then demonstrated that the gluon-to-quark splitting function ${\tilde{P}}_{qg}$ can be re-obtained through a simple extension of high energy factorized amplitudes to exact kinematics. Following this observation, two of us calculated in [2] the remaining splitting functions which involve quarks, ${\tilde{P}}_{gq}$, ${\tilde{P}}_{qg}$ and ${\tilde{P}}_{qq}$. In this paper we continue this program and compute the still missing TMD gluon-to-gluon splitting function ${\tilde{P}}_{gg}$. This requires a further modification of the formalism used in [2, 31], in particular when defining proper projection operators for high energy factorization. As a result we obtain a TMD gluon-to-gluon splitting function which agrees in the regarding limits not only with the DGLAP and BFKL limits, but also reduces in the soft limit to the Ciafaloni–Catani–Fiorani–Marchesini (CCFM) [48–50] splitting kernel.

The outline of this paper is as follows. In Sect. 2, we recall the definition of the TMD splitting functions and provide details about the steps for the calculation of their real contributions. Section 3 establishes an extension of the method previously used to derive gauge invariant 3-point vertices, and uses it to compute the corresponding 3-gluon vertex; the former used Lipatov’s effective action, the latter resorts to spinor helicity techniques. Section 4 is dedicated to a discussion of projection operators and their necessary modifications compared to Refs. [31, 32]. Sect. 5 contains the central results of this paper *i.e.* the complete set of real contributions to the TMD splitting functions. Sect. 6 is dedicated to a discussion of our results. Two appendices A and  B contain supplementary details and a representation of splitting kernels using an alternative set of variables.

## Definition of TMD splitting functions: real contributions

The matrix elements involved in the calculation of the real contributions to the leading order (LO) splitting functions are presented in Fig. 1. The incoming momentum, called *k*, features high energy kinematics, while the outgoing momentum, *q*, is taken in its most general form. The 4-momenta will be parametrised as follows

1 $$\begin{aligned}&k^\mu = y p^\mu + k_\perp ^\mu ,\nonumber \\&q^\mu = x p^\mu + q_\perp ^\mu + \frac{q^2+{ \varvec{q}}^2}{2x\,p\!\cdot \!n\,} n^\mu , \quad {\tilde{\varvec{q}}}= { \varvec{q}}- z {\varvec{k}}. \end{aligned}$$

Here *p* and *n* are two light-like momenta $\left( p^2 = n^2 = 0 \right)$ which refer to the two different light-cone directions for a fixed scattering axis; in the case of Deep-Inelastic-Scattering, one would for instance parametrize the virtual photon momentum as $q = n - x p$ with $x = Q^2/(2 p \cdot q)$, while *p* would yield the proton momentum in the limit of zero proton mass. We will also use $z=x/y$ to denote the longitudinal momentum fraction of the initial parton *k* carried on by the parton *q*. Within this setup, pure high energy kinematics corresponds to $z=0$, while collinear kinematics is obtained for ${\varvec{k}}=0$. Following the procedure outlined in [1, 2, 31], we start from the definition of the 2-particle irreducible (2PI) TMD kernel

2 $$\begin{aligned} {\hat{K}}_{ij} \left( z, \frac{{\varvec{k}}^2}{\mu ^2}, \epsilon , \alpha _s \right)= & {} z\, \int \frac{d q^2 d^{2 + 2 \epsilon } {{ \varvec{q}}}}{2 (2 \pi )^{4 + 2\epsilon }}\, \Theta (\mu _F^2 + q^2)\nonumber \\&\mathbb {P}_{j,\,\text {in}} \otimes \hat{K}_{ij}^{(0)}(q, k) \otimes \mathbb {P}_{i,\,\text {out}}, \end{aligned}$$

where $\hat{K}_{ij}^{(0)}$, $i,j=q,g$ denotes the squared matrix element for the transition of a parton *j* to a parton *i*, see Fig. 1, which includes the propagators of the outgoing lines. $\mathbb {P}_{j,\,\text {in}}$ and $\mathbb {P}_{j,\,\text {out}}$ are projectors, to be discussed in detail in Sect. 4. Gluons are taken in the $n\cdot A=0$ light-cone gauge $(n^2=0)$. The symbol $\otimes$ represents contraction of spin indices (see also Sect. 4); $\mu _F$ denotes the factorization scale, and we use dimensional regularisation in $d=4+2\epsilon$ dimensions with $\mu ^2$ the dimensional regularisation scale. It is convenient to introduce the following notation for the matrix element convoluted with the projectors,

3 $$\begin{aligned} g^2\, 2 \pi \, \delta \left( (k-q)^2\right) \, W_{ij} = \mathbb {P}_{j,\,\text {in}} \otimes \hat{K}_{ij}^{(0)}(q, k) \otimes \mathbb {P}_{i,\,\text {out}}, \end{aligned}$$

with

4 $$\begin{aligned} \delta \left( (k-q)^2\right)&= \frac{z}{1-z}\; \delta \left( q^2 + \frac{{\tilde{\varvec{q}}}^2 + z(1-z){\varvec{k}}^2}{1-z}\right) . \end{aligned}$$

With the $\overline{\text {MS}}$ strong coupling constant $\alpha _s = \frac{g^2 \mu ^{2\epsilon } e^{\epsilon \gamma _E}}{(4 \pi )^{1+ \epsilon }}$, and $\gamma _E$ the Euler-Mascheroni constant,

5 $$\begin{aligned} \frac{g^2}{2 (2 \pi )^{3 + 2\epsilon }} = \frac{\alpha _s}{4\pi } \frac{e^{-\epsilon \gamma _E}}{\pi ^{1+\epsilon }\mu ^{2\epsilon }}, \end{aligned}$$

and ${\tilde{\varvec{q}}}={ \varvec{q}}-z{\varvec{k}}$, Eq. (2) turns into

6 $$\begin{aligned} {\hat{K}}_{ij} \left( z, \frac{{\varvec{k}}^2}{\mu ^2}, \epsilon , \alpha _s \right)= & {} z\, \frac{\alpha _s}{4\pi }\, \frac{e^{-\epsilon \gamma _E}}{\mu ^{2\epsilon }}\nonumber \\&\times \int \frac{d^{2+2\epsilon }{\tilde{\varvec{q}}}}{\pi ^{1+\epsilon } \, {\tilde{\varvec{q}}}^2} \, \frac{z}{1-z}\, {\tilde{\varvec{q}}}^2\, W_{ij}\Big |_{q^2=-\frac{{\tilde{\varvec{q}}}^2 + z(1-z){\varvec{k}}^2}{1-z}} \nonumber \\&\times \Theta \left( \mu _F^2 - \frac{{\tilde{\varvec{q}}}^2 + z(1-z){\varvec{k}}^2}{1-z}\right) . \end{aligned}$$

This allows us to identify the transverse momentum dependent splitting function ${\tilde{P}}_{ij}^{(0)}$ as

7 $$\begin{aligned} {\tilde{P}}_{ij}^{(0)} (z, {\tilde{\varvec{q}}}, {\varvec{k}}) = \frac{z}{1-z}\, {\tilde{\varvec{q}}}^2\, \frac{1}{2} W_{ij}\Big |_{q^2=-\frac{{\tilde{\varvec{q}}}^2 + z(1-z){\varvec{k}}^2}{1-z}} \; , \end{aligned}$$

and we obtain

8 $$\begin{aligned} {\hat{K}}_{ij} \left( z, \frac{{\varvec{k}}^2}{\mu ^2}, \epsilon , \alpha _s \right)= & {} \frac{\alpha _s}{2\pi } \, z\, \frac{e^{-\epsilon \gamma _E}}{\mu ^{2\epsilon }} \nonumber \\&\times \int \frac{d^{2+2\epsilon }{\tilde{\varvec{q}}}}{ \pi ^{1+\epsilon } \,{\tilde{\varvec{q}}}^2} \, {\tilde{P}}_{ij}^{(0)} \nonumber \\&\times \Theta \left( \mu _F^2 - \frac{{\tilde{\varvec{q}}}^2 + z(1-z){\varvec{k}}^2}{1-z}\right) . \end{aligned}$$

With the angular averaged TMD splitting function defined as

9 $$\begin{aligned} \bar{P}_{ij}^{(0)} = \frac{1}{\pi } \int _0^{\pi } d\phi \,\sin ^{2\epsilon }\phi \; {\tilde{P}}_{ij}^{(0)} \end{aligned}$$

we finally arrive at

10 $$\begin{aligned} {\hat{K}}_{ij} \left( z, \frac{{\varvec{k}}^2}{\mu ^2}, \epsilon , \alpha _s \right)&= \frac{\alpha _s}{2\pi } z \frac{e^{-\epsilon \gamma _E}}{\Gamma (1+\epsilon )} \nonumber \\&\quad \times \frac{1}{2} \int _0^{(1-z)(\mu _F^2-z{\varvec{k}}^2)} \frac{d{\tilde{\varvec{q}}}^2}{{\tilde{\varvec{q}}}^2} \, \left( \frac{{\tilde{\varvec{q}}}^2}{\mu ^{2}}\right) ^\epsilon \nonumber \\&\quad \times \bar{P}_{ij}^{(0)}\left( z, \frac{{\varvec{k}}^2}{{\tilde{\varvec{q}}}^2} \right) . \end{aligned}$$

## Production vertices from spinor helicity amplitudes

The calculation of the real contributions to the ${\tilde{P}}_{qg}$, ${\tilde{P}}_{gq}$ and ${\tilde{P}}_{qq}$ in [2] was based on the effective 3-point vertices,

11 $$\begin{aligned} \Gamma ^\mu _{q^*g^*q}(q,k,p')= & {} i\,g\,t^a\, \left( \gamma ^\mu - \frac{n^\mu }{k\cdot n}\, q \! \! \! /\right) , \end{aligned}$$

12 $$\begin{aligned} \Gamma ^\mu _{g^*q^*q}(q,k,p')= & {} i\,g\,t^a\, \left( \gamma ^\mu - \frac{p^\mu }{p\cdot q}\, k \! \! \! /\right) , \end{aligned}$$

13 $$\begin{aligned} \Gamma ^\mu _{q^*q^*g}(q,k,p')= & {} i\,g\,t^a\, \left( \gamma ^\mu - \frac{p^\mu }{p\cdot p'}\, k \! \! \! /+ \frac{n^\mu }{n\cdot p'}\, q \! \! \! /\right) .\nonumber \\ \end{aligned}$$

These vertices have been obtained from Lipatov’s effective action formalism [33, 34] and afterwards slightly generalized to the TMD kinematics of Eq. (1). While a corresponding vertex $\Gamma _{g^*g^*g}$ can be easily obtain from Lipatov’s effective action, the generalization to TMD kinematics turns out to be far less trivial. Within the effective action formalism, an off-shell gluon corresponds to a reggeized gluon which is automatically associated with a specific polarization, proportional to the light-cone momenta *p* and *n*. While this is sufficient for the incoming off-shell gluon with momentum *k*, the CFP formalism requires open indices for the out-going gluon with momentum *q*. One is therefore driven to consider instead, the so-called gluon–gluon–reggeized gluon (GGR) vertex. This vertex is well known, see [34, 52] for a construction in covariant gauges. Indeed we will find that use of the corresponding GGR vertex in $A \cdot n= 0$ light-cone gauge is sufficient to calculate the TMD splitting kernel. Nevertheless the direct use of this vertex is not completely satisfactory: Within this vertex, the gluon with momentum *q* is treated as an ordinary QCD gluon; off-shellness of this gluon leads then naturally to a violation of current conservation and therefore gauge invariance. Below we verify that current conservation and therefore gauge invariance is restored by adding a term proportional to $n^\mu$, which is set to zero within the employed light-cone gauge. While such a restoration of current conservation might have been expected from the very beginning, we demonstrate below that such a term indeed arises out of a proper Feynman diagram analysis, deviating slightly from the strategy employed in [2]. In particular we demonstrate that the necessary production vertices can be as well obtained from a direct study of QCD scattering amplitudes in the high energy limit.

**Fig. 2:**
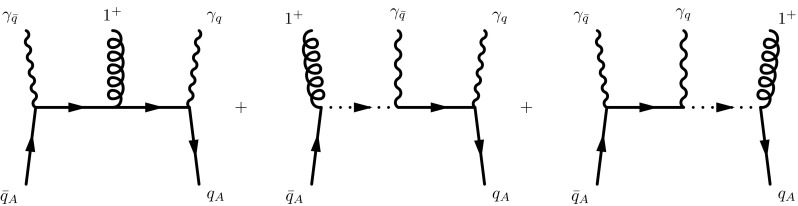
Feynman diagrams contributing to $\mathcal {A}(1^+,\bar{q}^{*+},q^{*-})$

To this end we will first recover the vertices (11)–(13) by stripping off the helicity dependence from scattering amplitudes computed by applying spinor helicity methods to high energy factorization [15, 16, 35–38]. In a next step we will use then this formalism to infer the structure of the 3-point gluon vertex to be used to compute ${\tilde{P}}_{gg}(z)$. While our result is obtained within the spinor helicity formalism, we would like to stress that this formalism provides merely a convenient framework for fast calculation; the obtained result is on the other hand completely general and could have been equally obtained through a study of QCD amplitudes using conventional Feynman rules. For an exhaustive reviews of the spinor helicity formalism, we refer the reader to [39–41].

While we finally aim at the TMD kinematics Eq. (1), the analysis in this section is at first limited to kinematics required by high energy factorization (see below for the precise parametrization used). The more general TMD kinematics Eq. (1) will then be addressed in Sect. 4.3. The reader who is only interested in the final form of the vertices can skip the rest of this section and just take for granted the formulae (11)–(13) for the 3-point vertices with a fermion pair, as well as the result (29) for the 3-gluon vertex.

To keep our derivation self contained, we recall the basic idea of the spinor helicity method to derive gauge invariant scattering amplitudes with off-shell particles [15, 35]: each off-shell particle is introduced into the diagram through an auxiliary on-shell pair; the squared sum of the on-shell momenta of the auxiliary pair accounts for the non vanishing squared momentum particle that they introduce. In the case of a gluon, a quark-antiquark pair is used; an off-shell quark is introduced via a vertex featuring an auxiliary photon-quark pair.^2^ The price to pay is the proliferation of Feynman diagrams, which are more than in the on-shell case; the additional diagrams feature propagators of the auxiliary particles. As demonstrated in [15], in the given kinematics the colour degrees of freedom of the pairs of auxiliary quarks are exactly equivalent to the colour of the corresponding off-shell gluon. On the other hand, since the auxiliary particles are on-shell, the gauge invariance of the resulting scattering amplitude is immediately manifest.

### Production by two off-shell quarks

We start with the simplest case, the production of an on-shell gluon by two off-shell fermions. By construction, amplitudes in the spinor helicity formalism are computed for specific values of the massless particle helicities [40]. This implies that the un-contracted off-shell vertex can be obtained from the amplitude by going backwards from the final result and stripping off the helicity dependence. The Feynman diagrams using the rules of [35] are given in Fig 2.

All the momenta are taken as incoming with

14 $$\begin{aligned} k_q= & {} x_q\,n + k_{q\perp }, \quad k_{\bar{q}} = x_{\bar{q}}\, p + k_{\bar{q}\perp }\nonumber \\ k_1= & {} p_1, \quad k_q + k_{\bar{q}} + p_1 = 0 \,, \end{aligned}$$

while $p_q = n$ and $p_{\bar{q}} = p$. Note that $k_q = -q|_{n\cdot q=0}$ and $k_{\bar{q}} = k$. In the last two diagrams of Fig. 2, the auxiliary fermions propagate eikonally through the diagrams; this is indicated with a dashed line. The resulting amplitude is then given by

15 $$\begin{aligned} \mathcal {A}(1^+,\bar{q}^{*+},q^{*-})= & {} \langle q| \frac{\epsilon \! \! \! /_{q\, +}}{\sqrt{2}} \frac{-k \! \! \! /_q}{k_q^2} \frac{\epsilon \! \! \! /_{1\,+}}{\sqrt{2}} \frac{k \! \! \! /_{\bar{q}}}{k_{\bar{q}}^2} \frac{\epsilon \! \! \! /_{\bar{q}\,-}}{\sqrt{2}} \nonumber \\&+\, \frac{\epsilon \! \! \! /_{q\,+}}{\sqrt{2}} \frac{k \! \! \! /_q}{k_q^2} \frac{\epsilon \! \! \! /_{\bar{q}\,-}}{\sqrt{2}} \frac{p \! \! \! /}{2 p\cdot k_q} \frac{\epsilon \! \! \! /_{1\,+}}{\sqrt{2}} + \nonumber \\&+\, \frac{\epsilon \! \! \! /_{1\,+}}{\sqrt{2}} \frac{n \! \! \! /}{2 n\cdot k_{\bar{q}}} \frac{\epsilon \! \! \! /_{q\,+}}{\sqrt{2}} \frac{k \! \! \! /_{\bar{q}}}{k_{\bar{q}}^2} \frac{\epsilon \! \! \! /_{\bar{q}\,-}}{\sqrt{2}} |\bar{q}]. \end{aligned}$$

In the first step we remove the polarization vector of the gluon $\epsilon _{1+}^{\mu }/{\sqrt{2}}$,^3^ in order to get rid of the dependency on the helicity of the on-shell gluon, and arrive at

**Fig. 3:**
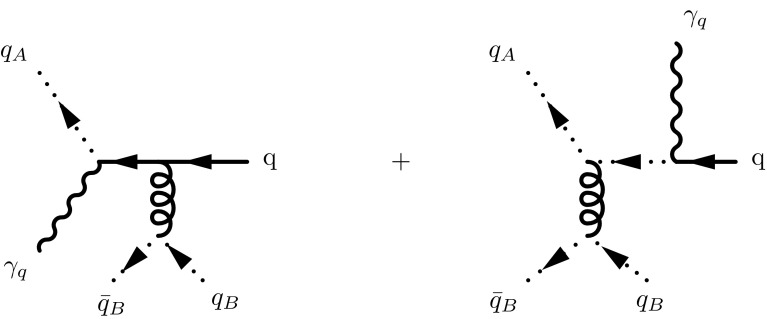
Feynman diagrams contributing to $\mathcal {A}(1^*,\bar{q}^{*+},q^-)$

16 $$\begin{aligned} \mathcal {A}(1^+,\bar{q}^{*+},q^{*-})\rightarrow & {} \frac{1}{2}\, \langle q| - \frac{ \epsilon \! \! \! /_{q\, +} \, k \! \! \! /_q \, \gamma ^\mu \, k \! \! \! /_{\bar{q}}\, \epsilon \! \! \! /_{\bar{q}\,-}}{k_q^2\, k_{\bar{q}}^2} \nonumber \\&+\, \frac{ \epsilon \! \! \! /_{q\,+} \, k \! \! \! /_q \, \epsilon \! \! \! /_{\bar{q}\,-} \, p \! \! \! /\, \gamma ^\mu }{k_q^2\, 2 p\cdot k_q} + \frac{\gamma ^\mu \, n \! \! \! /\, \epsilon \! \! \! /_{q\,+} \, k \! \! \! /_{\bar{q}} \, \epsilon \! \! \! /_{\bar{q}\,-} }{ 2 n\cdot k_{\bar{q}} \, k_{\bar{q}}^2} |\bar{q}].\nonumber \\ \end{aligned}$$

As the auxiliary photons are not observable, one should not keep track of their helicities, summing over them at the amplitude level. Nevertheless it turns out that there is only one choice which does not return a vanishing result. In particular, we can choose the momenta of the fermion pair, *n* and *p*, to be the auxiliary momenta necessary to build the polarization vector of each other’s auxiliary photon, *i.e.*

17 $$\begin{aligned} \epsilon ^\mu _{q\,+} = \frac{\langle \bar{q}| \gamma ^\mu |q]}{ \sqrt{2} \langle \bar{q}q\rangle }, \quad \epsilon ^\mu _{\bar{q}\,-} = \frac{\langle \bar{q}| \gamma ^\mu |q]}{ \sqrt{2} [\bar{q}q]}. \end{aligned}$$

In this way, when we explicitly open the slashed polarization vectors by using the spinor identity

18 $$\begin{aligned} \langle a| \gamma ^\mu c_\mu |b] = 2 \, \left( |a\rangle [b| + |b]\langle a| \right) \, , \end{aligned}$$

the first and third term of Eq. (16) vanish because $\langle p||p\rangle = [p||p] = 0$ for any massless momentum *p* and we obtain

19 $$\begin{aligned} - \frac{\langle \bar{q}| k \! \! \! /_{\bar{q}} \, \gamma ^\mu \, k \! \! \! /_{q} |q]}{k_q^2\,k_{\bar{q}}^2} + \frac{\langle \bar{q}|k \! \! \! /_q |q] \, p^\mu _{\bar{q}}}{k_q^2 \, p_{\bar{q}}\cdot k_{q}} + \frac{\langle \bar{q}|k \! \! \! /_{\bar{q}} |q] \, p^\mu _{q}}{k_{\bar{q}}^2 \, p_{q}\cdot k_{\bar{q}}} . \end{aligned}$$

This can be conveniently reshuffled as follows,

20 $$\begin{aligned} \langle \bar{q}| \frac{k \! \! \! /_{\bar{q}}}{k_{\bar{q}}^2} \, \left\{ \gamma ^\mu + \frac{p^\mu }{p\cdot k_q} k \! \! \! /_{\bar{q}} + \frac{n^\mu }{n\cdot k_{\bar{q}}} k \! \! \! /_q \right\} \, \frac{k \! \! \! /_q }{k_q^2} |q], \end{aligned}$$

and one can easily check that the term in curly brackets coincides with the vertex of Eq. (13) for the high energy kinematics Eq. (14).

### Production by an off-shell quark and an off-shell gluon

In the cases with one off-shell gluon, the contributing Feynman diagrams are shown in Fig. 3. The gluon propagator with momentum *k* will be now taken in light-cone gauge as

21 $$\begin{aligned} \frac{d_{\mu \nu }(k)}{k^2}, \quad \text {with} \quad d_{\mu \nu }(k) = -g_{\mu \nu } + \frac{k_\mu n_\nu + k_\nu n_\mu }{k\cdot n} . \end{aligned}$$

Again, all the momenta are taken as incoming and their parametrisation is

22 $$\begin{aligned} k_1= & {} x_1\,n + k_{1\perp },\quad k_{\bar{q}}= x_{\bar{q}}\, p + k_{\bar{q}\perp } ,\nonumber \\ k_q= & {} p_q , \quad k_1 + p_q + k_{\bar{q}} = 0, \end{aligned}$$

while $p_{\bar{q}} = p$ and $p_1 = n$; we also have $k_1 =- q|_{n \cdot q = 0}$ while $k_{\bar{q}} = k$. We find for the amplitude

23 $$\begin{aligned} \mathcal {A}(1^*,\bar{q}^{*-},q^{+})= & {} \frac{\langle 1|\gamma ^\mu |1]}{\sqrt{2}}\, \frac{d_{\mu \nu }(k_1)}{k_1^2} \, \langle \bar{q}| \frac{\epsilon \! \! \! /_{\bar{q}+}}{\sqrt{2}} \frac{k \! \! \! /_{\bar{q}}}{\bar{q}^2} \frac{\gamma ^\nu }{\sqrt{2}} \nonumber \\&- \frac{\gamma ^\nu }{\sqrt{2}} \frac{p \! \! \! /}{2p_q\cdot p}\, \frac{\epsilon \! \! \! /_{\bar{q}+}}{\sqrt{2}} |q]. \end{aligned}$$

After inserting the explicit expressions for the polarization vectors of the auxiliary photon (see “Appendix A”), we obtain

**Fig. 4:**
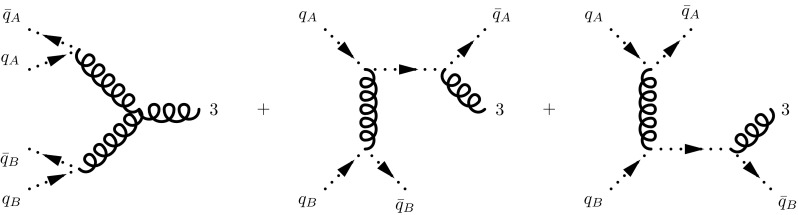
Feynman diagrams contributing to $\mathcal {A}(1^*,2^*,3^{\pm })$. Note that we have only three diagrams instead of the usual five diagrams found in the derivation of the Lipatov vertex. This is merely due to color ordering imposed on the helicity amplitudes and does not affect the final result

24 $$\begin{aligned} \mathcal {A}(1^*,\bar{q}^{*-},q^{+}) = p_1^\mu \, \frac{d_{\mu \nu }(k_1)}{k_1^2}\, [q| \, \left\{ \gamma ^\nu - \frac{p^\nu }{p \cdot p_q}\, k \! \! \! /_{\bar{q}} \right\} \, \frac{k \! \! \! /_{\bar{q}}}{k_{\bar{q}}^2} |\bar{q}],\nonumber \\ \end{aligned}$$

where the curly brackets contain the exact analogous of Eq. (12). The derivation of Eq. (11) follows then immediately. Note that the polarization tensor of the gluon propagator provides an overall factor. If this result had been obtained in a covariant gauge, this numerator would be

25 $$\begin{aligned} d^{\text {cov.}}_{\mu \nu }(k_1) = - g_{\mu \nu } + \xi \frac{k_{1\,\mu } k_{1\,\nu }}{k_1^2}. \end{aligned}$$

The contraction of the vertex in curly brackets with $k_{1\nu }$ vanishes, as can be easily checked by momentum conservation and the Dirac (Weyl) equation expressed in spinor helicity language $[q| p \! \! \! /_q = 0$, so that only $-g_{\mu \nu }$ survives for a covariant gauge. This makes it irrelevant (up to a sign) to include or not the numerator in the factor outside the curly brackets. In the light-cone gauge, this is no longer the case, since the polarization tensor Eq. (21) is not invertible. Thus, the contraction of $n_\nu$ with the bracketed vertex does not vanish and the equivalence with the covariant gauge is lost, at the pure amplitude level. This does not have physical consequences, since the amplitude is not an observable. It might however lead to an ambiguity for the computation of the splitting function, because the two expressions for the off-shell vertex,

26 $$\begin{aligned} \left\{ \gamma ^\nu - \frac{p_{\bar{q}}^\nu }{p_{\bar{q}} \cdot p_q}\, k \! \! \! /_{\bar{q}} \right\} \quad \text {vs.} \quad \frac{d_{\mu \nu }(k_1)}{k_1^2}\, \, \left\{ \gamma ^\nu - \frac{p_{\bar{q}}^\nu }{p_{\bar{q}} \cdot p_q}\, k \! \! \! /_{\bar{q}} \right\} ,\nonumber \\ \end{aligned}$$

could lead to different splitting functions, when the CFP procedure is applied [2, 32]. Nevertheless such an ambiguity does not arise. First, because including the numerator of the gluon propagator is necessary in order to be consistent (as we will demonstrate in the next section). Second, because, when working completely consistently in light-cone gauge, a modified spin projector for the incoming gluons is necessary in order to apply the CFP procedure to off-shell matrix elements, as we will discuss extensively in Sect. 4. This completely eliminates any ambiguity and yields a result identical to the one obtained previously for ${\tilde{P}}_{qg}$ by using the vertex without the numerator of the gluon propagator [2].

### Production by two off-shell gluons

The three Feynman diagrams for an amplitude with 2 off-shell and one on-shell gluons are depicted in Fig. 4: the first piece is the ordinary QCD 3-gluon vertex augmented by the propagators of the off shell gluons with the corresponding couplings to the auxiliary fermion pairs; then there are the two extra contributions in which the auxiliary quark lines eikonalize. The expression for the off-shell 3-gluon amplitude in terms of the three depicted contributions is given in [36, 42]. Here we provide only the result after factoring out the denominators of the off-shell gluon propagators with the momenta

27 $$\begin{aligned} k_1 = x_1\,p + k_{1\perp }, \quad k_{2} = x_2\, n + k_{2\perp }, \quad k_1 + k_2 + p_3 = 0,\nonumber \\ \end{aligned}$$

and $\mathcal {V}^{\mu _1\mu _2\mu _3}(k_1,k_2,p_3)$ the ordinary QCD three-gluon vertex, one has

28 $$\begin{aligned} \mathcal {A}(g_1^*,g_2^*,g_3)= & {} \sqrt{2}\, \frac{p_{ \mu _1} \,n_{\mu _2}\, \epsilon _{\mu _3}(p_3)}{k_1^2 \, k_2^2}\nonumber \\&\times \,\bigg \{ \mathcal {V}^{\lambda \kappa \mu _3}(k_1,k_2,p_3) \, {d^{\mu _1}}_{\lambda } (k_1)\, {d^{\mu _2}}_{\kappa }(k_2) \nonumber \\&+\, d^{\mu _1\mu _2}(k_2)\, \frac{k_1^2 p^{\mu _3}}{p\cdot p_3} - d^{\mu _1\mu _2}(k_1)\, \frac{k_2^2 n^{\mu _3}}{n\cdot p_3} \bigg \}.\nonumber \\ \end{aligned}$$

In [36] the computations were performed in the Feynman gauge, with $d^{\text {cov.}}_{\mu \nu }(k) = - g_{\mu \nu }$. Here we use instead the light-cone gauge polarization tensor Eq. (21). Due to gauge invariance, the only difference in the construction of the vertex is a change in the gluon polarization tensor. Note that since the light-cone gauge polarization tensor $d_{\mu \nu }(k)$, Eq. (21), is not invertible, it is not possible to extract the numerators of both gluon propagators simultaneously for all the three terms in the curly brackets in Eq. (28). We therefore retain these polarization tensors in the expression for the off-shell vertex.

The above vertex, extracted for high energy kinematics Eq. (27) is then used to construct the corresponding vertex for TMD kinematics Eq. (1). The final form of the gluon production vertex in high energy kinematics, using the naming convention for momenta as in Eqs. (11–13) and (1), is given by

29 $$\begin{aligned} \Gamma ^{\mu _1\mu _2\mu _3}_{g^*g^*g}(q,k,p')= & {} \mathcal {V}^{\lambda \kappa \mu _3}(-q,k,-p') \, {d^{\mu _1}}_{\lambda } (q)\, {d^{\mu _2}}_{\kappa }(k) \nonumber \\&+\, d^{\mu _1\mu _2}(k)\, \frac{q^2 n^{\mu _3}}{ n\cdot p'}\nonumber \\&-\, d^{\mu _1\mu _2}(q)\, \frac{k^2 p^{\mu _3}}{ p\cdot p'}, \nonumber \\ \text {with}\,\, n\cdot k= & {} n \cdot p' \quad p\cdot q = -p\cdot p'. \end{aligned}$$

As indicated in the last line of the above expression, this result is so far only valid for the special case $n\cdot k=n \cdot p'$ since $q \cdot n = 0$ in high energy kinematics. TMD kinematics Eq. (1) requires on the other hand $q \cdot n \ne 0$ which breaks the symmetry of the second term of Eq. (29) under exchange of momenta $k \leftrightarrow p'$. We will find that the form already given in Eq. (29) provides finally the correct generalization to TMD kinematics (since it guarantees current conservation for the produced gluon). However, in order to demonstrate this we need to make use of the structure of the modified projectors, which will be discussed in the following section.

## Modifying the collinear projectors

As a next step, we introduce the CFP approach to collinear factorization, explain the role of the projectors and motivate the need for the modifications which allow to consistently accommodate them in our framework. To make the present discussion as self-contained as possible, we first remind the basics which motivated their introduction in the case of collinear factorization [32, 43], following closely Sect. 2.2 of [31].

### Collinear case

The underlying concept is based on the observation that the hard matrix element contains collinear divergences which can be factorised, to all orders in $\alpha _s$, and reabsorbed into a process-independent transition-functions $\Gamma$. Calling $\sigma ^{(0)}$ the leading-twist contribution to the inclusive cross section, this can be schematically written as

30 $$\begin{aligned} \sigma ^{(0)} = C\, \Gamma , \end{aligned}$$

where *C* is the renormalized and finite (for $\epsilon \rightarrow 0$) hard matrix element, $\Gamma$ is the transition function which absorbs only the collinear poles in $1/\epsilon$ and a momentum integration is implied. The transition function can then be used to introduce the parton density functions,

31 $$\begin{aligned} {\tilde{f}}(x,\mu _F) \equiv \Gamma \, {\tilde{f}}^{(0)}(x,\mu _F), \end{aligned}$$

leading to the usual factorisation formula. The possibility to perform such a factorisation was proven in [32, 43], where use of the light-cone gauge turned out to be crucial.^4^ The first result of [43] is that the cross section can be expanded as a series of 2PI (2-Particle-Irreducible) kernels $K^{(0)}$, describing the parton evolution before the hard scattering, times a hard scattering coefficient function $C^{(0)}$,

32 $$\begin{aligned} \sigma ^{(0)}= & {} C^{(0)} \, \bigg ( 1 + K^{(0)} + K^{(0)}\, K^{(0)} + \cdots \bigg ) \equiv C^{(0)} \, \mathcal {G}^{(0)}, \nonumber \\ \mathcal {G}^{(0)}\equiv & {} \bigg ( 1 + K^{(0)} + K^{(0)}\, K^{(0)} + \cdots \bigg ) = \frac{1}{1- K^{(0)}}\, . \end{aligned}$$

**Fig. 5:**
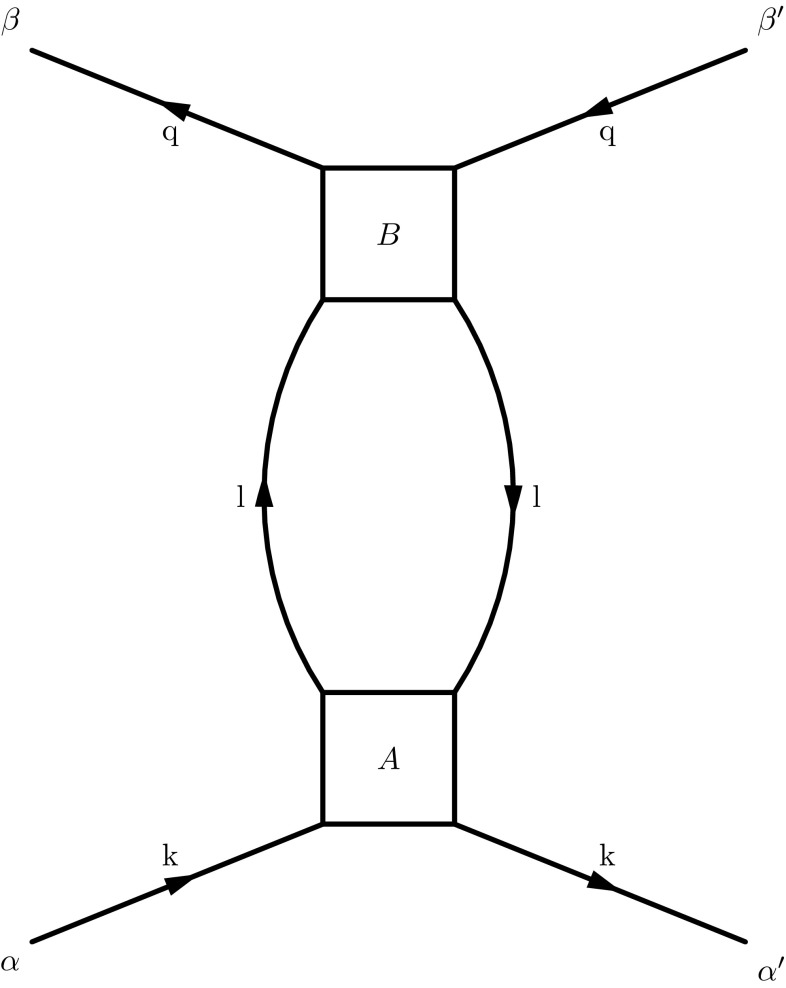
Convolution of two generic 2PI amplitudes *A* and *B*. Notice that the indices $\alpha$, $\beta$ and $\gamma$ maybe spinor as well as Lorentz indices, depending on the particles going in and coming out of the kernels. The whole kernel, which we can denote by *C*, is given by the convolution $C^{\alpha \alpha '}_{\beta \beta '}(k,q) = \int \frac{d^n l}{(2\pi )^n}\, A^{\alpha \alpha '}_{\gamma \gamma '}(k,l)\, B^{\gamma \gamma '}_{\beta \beta '}(l,q)$

By definition, each 2PI kernel $K^{(0)}$ (see Fig. 5 for an illustration of two linked 2PI kernels) has propagators only in the upper legs, which are connected by a momentum integral, $\int d^d l$, with the lower legs of the following 2PI kernel all the way up to the coefficient function. The central result of [43] is that the kernels themselves are free from infrared singularities in the light-cone gauge, such that all the collinear poles in $1\over \epsilon$ come from the integration over the intermediate momenta connecting the kernels. Then, one introduces a projector $\mathbb {P}_C$ which isolates the infrared poles of $K^{(0)}$ [32],

33 $$\begin{aligned} K^{(0)} = \left( 1 - \mathbb {P}_C \right) \, K^{(0)} + \mathbb {P}_C\, K^{(0)}, \end{aligned}$$

in such a way that the second term contains all the singularities, whereas the first is free of poles in $\epsilon$. Factorization emerges when one notices that (33) can be used recursively to reorganise the expansion of the process-independent Green function (32) as

34 $$\begin{aligned} \mathcal {G}^{(0)}&= \mathcal {G} \, \Gamma , \quad \mathcal {G} = \frac{1}{1-(1-\mathbb {P}_C)\,K^{(0)} } , \end{aligned}$$

35 $$\begin{aligned} \Gamma&= \frac{1}{1- \mathbb {P}_C \,K } \nonumber \\&= 1 + \mathbb {P}_C \,K + \mathbb {P}_C \, \left[ K\, \mathbb {P}_C \,K \right] + \cdots , \quad K \equiv K^{(0)} \, \mathcal {G}, \end{aligned}$$

where the squared brackets are introduced to highlight that projectors operate on everything to their right. Since (34) is clearly free of singularities, it is apparent that we have sketched how to arrive at Eq. (30), setting

36 $$\begin{aligned} C \equiv C^{(0)} \, \mathcal {G}. \end{aligned}$$

To further understand the projectors, we split them into one projector acting on the spin indices and one acting on the momentum space integral between two consecutive kernels,

37 $$\begin{aligned} \mathbb {P}_C \equiv \mathbb {P}^{\epsilon } \otimes \mathbb {P}^{s}. \end{aligned}$$

Here, the momentum space projector $\mathbb {P}^{\epsilon }$ extracts the singular part of the integral over the intermediate momentum, transforming it into a convolution integral, whereas $\mathbb {P}^{s}$ decouples lower and upper kernel in the spin indices. It is apparent that $\mathbb {P}^{\epsilon }$ is independent of the particle species propagating through the 2PI ladder, whereas $\mathbb {P}^{s}$ is closely related to it. In [32] an explicit argument is presented which identifies the proper projector for the non-singlet sector, whereas [45] presents the projector if two 2PI kernels are connected by a gluon. Referring to Fig. 5 for our nomenclature of the kernels and the momenta, we have38when the intermediate particle is a quark, with indices belonging to $\gamma$-matrices, and

39 $$\begin{aligned}&A(q,l)_{\dots \mu '\nu '}\, \mathbb {P}^{s\, \mu '\nu '}_{\,\,\, \mu \nu }\, B^{\dots \mu \nu }(l,k) \nonumber \\&\quad \equiv A(q, l)_{\dots \mu '\nu '}\frac{d^{\mu '\nu '}(l)}{d-2}\, (- g_{\mu \nu })\, B^{\dots \mu \nu }(l,k), \end{aligned}$$

if the intermediate particle is a gluon with Lorentz tensor indices; *d* specifies the number of space-time dimensions. We can further split the spin projectors into an “in” and “out” component

40 $$\begin{aligned} \mathbb {P}^{s} \equiv \mathbb {P}^{s}_{\text {in}} \otimes \mathbb {P}^{s}_{\text {out}}. \end{aligned}$$

The names “in” and “out” can be understood from Fig. 5 and interpreting the diagram in terms of a parton evolution unfolding upwards. In other words, the amplitude *A* represents a series of parton emissions from an initial parton with momentum *k* which emerges with a momentum *l* and then undergoes another series of splittings represented by the kernel B. The spin projectors for the collinear case can be summarised, for the gluon and quark case respectively, as41where we have omitted the spinor indices for the sake of brevity, as we will be doing through the rest of this paper. Two comments are in order here:

1. The incoming projectors yield the average over the gluon or quark helicities in *d* dimensions.
2. The projectors are not uniquely defined: both the momentum projector $\mathbb {P}^{\epsilon }$ (due to the arbitrariness of the factorization scheme) and the spin projectors are defined modulo finite terms and a re-definition is possible as long as all singular terms are properly extracted.

For the sake of completeness, we further report here the action of the whole projector on the product of two kernels in the case of an intermediate quark state, in order to clarify the role of the momentum space projector $\mathbb {P}^\epsilon$; it is explicitly given by

42 $$\begin{aligned} A \, \mathbb {P}_C \,B\equiv & {} A \, \mathbb {P}^\epsilon \otimes \mathbb {P}^s \,B = A(q, l)_{\dots \alpha \alpha '}\, \frac{(l \! \! \! /)^{\alpha \alpha '}}{2}\, \mathbb {P}^{\epsilon }\, \int \frac{d^ml}{(2\pi )^m}\nonumber \\&\times \, \frac{(n \! \! \! /)_{\beta \beta '}}{2n\cdot l}\, B^{\dots \beta \beta '}(l,k), \end{aligned}$$

where $$\mathbb {P}^\epsilon $$ sets $$l^2 = 0 $$ in the *A* kernel and takes the pole part (or pole part plus finite terms, depending on the scheme choice) of the integral over $$d^d l \propto dl^2\, d^{d-2}l_\perp $$, with the integral over $$l^2$$ defined up to the factorization scale.^5^ The reason for setting $$l^2 = 0$$ in the left-hand kernel is clear in light of the proof of [43] that the infrared singularities for a cross section which is 2PI expanded in light-like gauge come only from the integral over the intermediate propagator momenta, which, by definition, are attached only to the outgoing lines of the kernels.

### Generalization to the TMD case

In the case where the incoming particle has a transverse momentum component parametrised as in (1), an economic choice for the projectors has been introduced by Catani and Hautmann in [31] (gluon case) and two of us in [2] (quark case):43where the difference to the collinear case Eq. (43) lies in the incoming projectors. While for the quark it is easy to see that the numerator of the incoming projector merely provides the collinear part of the momentum dotted into the gamma matrices (and actually coincides completely with the collinear case), for the gluon both projectors are at first considerably different. While such polarizations for high energy factorized gluons are well known in the literature and can be traced back to a gauge rotation of the original ‘non-sense’ polarization $\sim p^\mu , n^\mu$ of a reggeized gluon (see [47] for a pedagogic introduction) it is interesting to take a closer look at the precise motivation given by the authors of [31], which essentially refers to [13]. In a nutshell, [13] considers the amplitude for heavy quark pair production via the fusion of two off-shell gluons. As outlined in the “Appendix B” of [13], this amplitude is defined to contain the polarization tensor in axial gauge for each of two off-shell gluons. The open gluon indices are then contracted by ‘non-sense’ polarization vectors $\sim p^\mu$ and $\sim n^\mu$, as it is adequate for high energy factorized gluons. The crucial observation is then, that contraction of the polarization tensor in axial gauge with ‘non-sense’ polarization vectors provides a vector proportional to the transverse momentum of the regarding gluon. In our case this relation is encoded in the identity

44 $$\begin{aligned} y\, p^\mu \, d_{\mu \nu }(k) = k_{\perp \,\nu } \qquad \text {if} \qquad k^\mu = y\,p^{\mu } + k^\mu _\perp \, . \end{aligned}$$

In turn, it is then possible to simply work with external off-shell gluonic legs with the axial gauge tensor of the external gluonic legs already stripped off. However, in our case the scenario is slightly different: since the generalized production vertex requires the presence of the polarization tensors in axial gauge and these cannot be factorized, we must simply contract the open index of our initial high energy factorized gluon with $y p^\mu$, as suggested by the original formulation of high energy factorization, with no possibility for further simplifications. However, doing so seems to lose one of initial advantage of the choice $k_\perp ^\mu k_\perp ^\nu / k_\perp ^2$, i.e. that, using the same “out” projector as for the collinear case, the resulting modified projector

$$\begin{aligned} \mathbb {P}^s_g = -\frac{k_\perp ^{\mu '} k_\perp ^{\nu '}}{k_\perp ^2} \, (-g_{\mu \nu }), \end{aligned}$$

satisfies the necessary constraints of being equal to its square and reducing to the collinear projector after integration over the azimuthal angle, in the limit of vanishing transverse momentum

45 $$\begin{aligned} \mathbb {P}^s_{g} \otimes \mathbb {P}^s_{g}= & {} \frac{k_\perp ^{\mu '}\, k_\perp ^{\nu '}}{k_\perp ^2} \, (-g_{\mu \nu }) \, \frac{k_\perp ^{\mu }\, k_\perp ^{\nu }}{k_\perp ^2} \, (-g_{\rho \sigma })\nonumber \\= & {} -\frac{k_\perp ^{\mu '}\, k_\perp ^{\nu '}}{k_\perp ^2} \, (-g_{\rho \sigma })= \mathbb {P}^s_{g}, \end{aligned}$$

46 $$\begin{aligned} \left\langle \frac{k_\perp ^{\mu }\, k_\perp ^{\nu }}{k_\perp ^2} \right\rangle _\phi&{\mathop {=}\limits ^{k_\perp \rightarrow 0}}&\frac{d^{\mu \nu }(k = z\,p)}{d-2}. \end{aligned}$$

Since for the 3-gluon vertex $\Gamma ^{\mu _1\mu _2\mu _3}_{g_1^*g_2^* g_3}$ it is not possible to factor out the numerators of the gluon propagators, the choice of the Catani-Hautmann projector is unfeasible for our purposes, which makes

47 $$\begin{aligned} \mathbb {P}^s_{g\, \text {in}} = -y^2\, \frac{p^{\mu } p^{\nu }}{k_\perp ^2} \end{aligned}$$

the natural choice for the incoming projector. However, in order for (45) to hold, we cannot keep the same “out” projector. It is nevertheless possible to use the projector proposed already in [2],

48 $$\begin{aligned} \mathbb {P}^s_{g\, \text {out}} = -g^{\mu \nu } + \frac{k^\mu n^\nu + k^\nu n^\mu }{k\cdot n} - k^2\, \frac{n_\mu n_\nu }{(k\cdot n)^2}\, . \end{aligned}$$

It is easy to check that this restores the condition (45), $\mathbb {P}^s\otimes \mathbb {P}^s = \mathbb {P}^s$. Moreover, as already noted in [2] the new “out” projector, by itself, is consistent with the collinear case. In an actual calculation all terms apart from the $-g_{\mu \nu }$ are set to zero by the polarization tensor of the gluon propagators connecting to the projector, due to the property $d_{\mu \nu } \cdot n^\nu = 0 = d_{\mu \nu } \cdot n^\mu$. We explicitly checked that changing the “out” projector in the collinear case does not modify the LO collinear splitting functions.

What remains to be checked is that the collinear limit holds also for the “in” projector. Since the numerator is purely longitudinal, there exists no relation similar to Eq. (46) after angular averaging, at least not at the general operator level. However, it can be proved that the difference between $y^2\, p^\mu p^\nu /k_\perp ^2$ and $k^\mu _\perp k^\nu _\perp /k_\perp ^2$ vanishes when they are contracted into the relevant vertices.

We have to show this for two vertices, $\Gamma ^\mu _{q^*g^*q}(q,k,p')$, (for the ${\tilde{P}}_{qg}$ kernel), and $\Gamma ^{\mu _1\mu _2\mu _3}_{g^*g^*g}(k,q,p')$, (for ${\tilde{P}}_{gg}$).

The first case is trivial since due to the factor $d^{\mu \nu }(k)$, the projector $y^2\, p^\mu p^\nu / k_\perp ^2$ automatically reduces to $k_\perp ^\mu k_\perp ^\nu / k_\perp ^2$ due to Eq. (44).

As for $\Gamma ^{\mu _1\mu _2\mu _3}_{g^*g^*g}(k,q,p')$, it is easy to check that

49 $$\begin{aligned} k_{\mu _1}\, \Gamma ^{\mu _1\mu _2\mu _3}_{g^*g^*g}(k,q,p') = \mathcal {O}(k_\perp ^2). \end{aligned}$$

As a consequence we find

50 $$\begin{aligned} y\, p_{\mu _1}\, \Gamma ^{\mu _1\mu _2\mu _3}_{g^*g^*g}(k,q,p')&= - k_{\perp \, \mu _1}\, \Gamma ^{\mu _1\mu _2\mu _3}_{g^*g^*g}(k,q,p')\nonumber \\&\quad + \mathcal {O}(k_\perp ^2) \, , \end{aligned}$$

which is sufficient to establish agreement in the collinear limit, at least at the perturbative order which we are considering; indeed, this means that the collinear limit is exactly the same as for the Catani-Hautmann projector. Therefore, the final set of projectors which we will use in the following is given by51Finally, let us note that the convolution product we have just dissected in detail is precisely the one in Eq. (2) from which we derived the definition of our TMD splitting functions. For the collinear case, an all-order argument for the derivation of splitting functions is presented in [32], to which we refer the interested reader.

### Gauge invariance of the effective production vertex

The effective gluon production vertex,

52 $$\begin{aligned} \Gamma ^{\mu _1\mu _2\mu _3}_{g^*g^*g}(q,k,p')&= \mathcal {V}^{\lambda \kappa \mu _3}(-q,k,-p') \, {d^{\mu _1}}_{\lambda } (q)\, {d^{\mu _2}}_{\kappa }(k) \nonumber \\&\quad + d^{\mu _1\mu _2}(k)\, \frac{q^2 n^{\mu _3}}{ n\cdot p'} - d^{\mu _1\mu _2}(q)\, \frac{k^2 p^{\mu _3}}{ p\cdot p'} \end{aligned}$$

obtained in Eq. (29) is so far still restricted to pure high energy kinematics, where $n \cdot q = 0$ and therefore $n \cdot k = n \cdot p'$. This no longer holds for the more general TMD kinematics Eq. (1). While the QCD three gluon vertex is already fixed for general momenta, $n \cdot k \ne n \cdot p'$ leaves us at first with an ambiguity for the denominator of the second term in Eq. (29) which cannot be fixed by high energy factorization alone. Similar to the case of the quark splitting functions, we find that this ambiguity can be solved if we require current conservation for the produced real gluon. To this end we first recall the Ward identity of the QCD three gluon vertex in the case where both gluons are off-shell,

53 $$\begin{aligned} \mathcal {V}^{\lambda \kappa \mu _3}(-q,k,-p') \cdot p'_{\mu _3}&= \left( k^{\lambda } k^{\kappa } - q^\lambda q^\kappa \right) + \left( q^2 - k^2 \right) g^{\lambda \kappa }\, . \end{aligned}$$

Adding polarization tensors and contraction with the polarization of the incoming (high energy factorized) gluon, $y p_{\mu _2}$, one has

54 $$\begin{aligned}&\mathcal {V}^{\lambda \kappa \mu _3}(-q,k,-p') \cdot p'_{\mu _3} \cdot d^{\mu _1}{}_{\lambda }(q) d^{\mu _2}{}_\kappa y p_{\mu _2} \nonumber \\&\quad = k^2 ( d^{\mu _1}{}_{\lambda }(q ) y p^\lambda ) - q^2 ( d^{\mu _1}{}_{\lambda }(k ) y p^\lambda ). \end{aligned}$$

On the other hand one finds for the sum of non-local terms

55 $$\begin{aligned}&y\,p_{\mu _2} \left( d^{\mu _1\mu _2}(k)\, \frac{q^2 n^{\mu _3}}{ n\cdot p'} - d^{\mu _1\mu _2}(q)\, \frac{k^2 p^{\mu _3}}{ p\cdot p'} \right) \cdot p_{\mu _3}' \nonumber \\&\quad = y\,p_{\mu _2} \bigg ( d^{\mu _1\mu _2}(k)\, q^2 - d^{\mu _1\mu _2}(q)\, k^2 \bigg ), \end{aligned}$$

which cancels precisely the terms arising in Eq. (54) and we demonstrated current conservation with respect to the produced gluon. Note that this is only achieved if we fix the denominator of the second term in Eq. (52) to the form given above.

### Comparison to Lipatov’s effective action

At this stage we finally return to the discussion at the beginning of Sect. 3. Calculating the gluon-gluon-reggeized gluon (GRR) vertex in $A \cdot n = 0$ light-cone gauge from Lipatov’s high energy effective action, where the reggeized gluon is identified with the incoming gluon with momentum *k* and the gluons with momenta *q* and $p'$ are treated as conventional QCD gluons, one finds

56 $$\begin{aligned}&\Gamma _{GGR}^{\lambda \mu _3}(q,k,p') \propto p_{\mu _2} d^{\mu _2}_{\kappa }(k) \mathcal {V}^{\lambda \kappa \lambda }(-q,k,-p') \nonumber \\&\quad - p^{\lambda } p^{\mu _3}\frac{k^2}{ p\cdot p'}, \end{aligned}$$

where ‘$\propto$’ merely serves to indicate that the above expression does not coincide with the overall normalization as provided within Lipatov’s effective action. Adding the polarization tensor of the gluon with momentum *q* to this vertex, one finds easily

57 $$\begin{aligned}&d^{\mu _1}{}_\lambda (q) \Gamma _{GGR}^{\lambda \mu _3}(q,k,p') \nonumber \\&\quad \propto p_{\mu _2}\left[ \mathcal {V}^{\lambda \kappa \lambda }(-q,k,-p') d^{\mu _2}_{\kappa }(k) d^{\mu _1}{}_\lambda (q) - d^{\mu _2}{}_\lambda (q) \frac{k^2 p^{\mu _3}}{p \cdot p'} \right] . \end{aligned}$$

Since within light-cone gauge the term proportional to $n^{\mu _3}$ in Eq. (52) will be set to zero, use of the above expression is equivalent to the use of Eq. (52) within the employed modification of the CFP formalism. However, due to the absence of the term proportional to $n^{\mu _3}$, a direct verification of gauge invariance is not possible.

## Results for the real emission splitting functions

In this section we present our results for the TMD splitting functions. When performing the calculations, we follow exactly the steps that are outlined in Sect. 2, using the modified vertices and projectors defined in Sects. 3 and 4. We discuss in more detail the case of the ${\tilde{P}}_{gg}$ kernel, as this is the first time this TMD splitting functions is being calculated using a CFP inspired framework.

### Quark splitting functions

For the quark splitting functions previously computed in [1, 2, 31] we confirm the previous results, after including the modification discussed in the foregoing section. For completeness we present here the precise expressions for the TMD splitting functions

58 $$\begin{aligned} {\tilde{P}}_{qg}^{(0)}= & {} T_R\, \left( \frac{{\tilde{\varvec{q}}}^2}{{\tilde{\varvec{q}}}^2+z(1-z){\varvec{k}}^2}\right) ^2 \nonumber \\&\times \left[ 1 + 4z^2(1-z)^2\frac{{\varvec{k}}^2}{{\tilde{\varvec{q}}}^2} + 4z(1-z)(1-2z)\frac{{\varvec{k}}\cdot {\tilde{\varvec{q}}}}{{\tilde{\varvec{q}}}^2} \right. \nonumber \\&\left. - 4z(1-z)\frac{({\varvec{k}}\cdot {\tilde{\varvec{q}}})^2}{{\varvec{k}}^2{\tilde{\varvec{q}}}^2} \right] , \end{aligned}$$

59 $$\begin{aligned} {\tilde{P}}_{gq}^{(0)}= & {} C_F\, \left( \frac{{\tilde{\varvec{q}}}^2}{{\tilde{\varvec{q}}}^2+z(1-z){\varvec{k}}^2}\right) ^2\, \frac{{\tilde{\varvec{q}}}^2}{({\tilde{\varvec{q}}}-(1-z){\varvec{k}})^2} \nonumber \\&\times \left[ \frac{2}{z} - 2 + z + 2(1-z)(1+z-z^2)\frac{{\varvec{k}}^2}{{\tilde{\varvec{q}}}^2} + z(1-z)^2(1+z^2)\frac{{\varvec{k}}^4}{{\tilde{\varvec{q}}}^4}\right. \nonumber \\&\left. +\, 4z^2(1-z)^2\frac{{\varvec{k}}^2 \, {\varvec{k}}\cdot {\tilde{\varvec{q}}}}{{\tilde{\varvec{q}}}^4} + 4(1-z)^2\frac{{\varvec{k}}\cdot {\tilde{\varvec{q}}}}{{\tilde{\varvec{q}}}^2} + 4z(1-z)^2\frac{({\varvec{k}}\cdot {\tilde{\varvec{q}}})^2}{{\tilde{\varvec{q}}}^4} \right] \nonumber \\&+\, \epsilon \, C_F\, \frac{z{\tilde{\varvec{q}}}^2 \left( {\tilde{\varvec{q}}}-(1-z){\varvec{k}}\right) ^2}{({\tilde{\varvec{q}}}^2+z(1-z){\varvec{k}}^2)^2}, \end{aligned}$$

60 $$\begin{aligned} {\tilde{P}}_{qq}^{(0)}= & {} C_F\, \left( \frac{{\tilde{\varvec{q}}}^2}{{\tilde{\varvec{q}}}^2+z(1-z){\varvec{k}}^2}\right) ^2 \frac{{\tilde{\varvec{q}}}^2}{({\tilde{\varvec{q}}}-(1-z){\varvec{k}})^2} \nonumber \\&\times \left[ \frac{1+z^2}{1-z} + (1+z+4z^2-2z^3)\frac{{\varvec{k}}^2}{{\tilde{\varvec{q}}}^2} + z^2(1-z)(5-4z+z^2)\frac{{\varvec{k}}^4}{{\tilde{\varvec{q}}}^4}\right. \nonumber \\&\left. +\, 2z(1-2z)\frac{{\varvec{k}}\cdot {\tilde{\varvec{q}}}}{{\tilde{\varvec{q}}}^2} + 2z(1-z)(1-2z)(2-z)\frac{{\varvec{k}}^2\, {\varvec{k}}\cdot {\tilde{\varvec{q}}}}{{\tilde{\varvec{q}}}^4}\right. \nonumber \\&\left. - \, 4z(1-z)^2\frac{({\varvec{k}}\cdot {\tilde{\varvec{q}}})^2}{{\tilde{\varvec{q}}}^4} \right] + \epsilon \, C_F\, \frac{(1-z){\tilde{\varvec{q}}}^2({\tilde{\varvec{q}}}+z{\varvec{k}})^2}{({\tilde{\varvec{q}}}^2+z(1-z){\varvec{k}}^2)^2}, \end{aligned}$$

and for the angular averaged TMD splitting functions (with $\epsilon =0$)

61 $$\begin{aligned} \bar{P}_{qg}^{(0)}= & {} T_R \left( \frac{{\tilde{\varvec{q}}}^2}{{\tilde{\varvec{q}}}^2+z(1-z){\varvec{k}}^2}\right) ^2\, \left[ z^2 + (1-z)^2 + 4z^2(1-z)^2\frac{{\varvec{k}}^2}{{\tilde{\varvec{q}}}^2} \right] , \end{aligned}$$

62 $$\begin{aligned} \bar{P}_{gq}^{(0)}= & {} C_F\, \left[ \frac{2{\tilde{\varvec{q}}}^2}{z|{\tilde{\varvec{q}}}^2-(1-z)^2{\varvec{k}}^2|} - \frac{(2-z){\tilde{\varvec{q}}}^4+z(1-z^2){\varvec{k}}^2{\tilde{\varvec{q}}}^2}{\left( {\tilde{\varvec{q}}}^2+z(1-z){\varvec{k}}^2\right) ^2} \right] , \end{aligned}$$

63 $$\begin{aligned} \bar{P}_{qq}^{(0)}= & {} C_F\, \frac{{\tilde{\varvec{q}}}^2}{{\tilde{\varvec{q}}}^2+z(1-z){\varvec{k}}^2} \nonumber \\&\times \left[ \frac{{\tilde{\varvec{q}}}^2+(1-z^2){\varvec{k}}^2}{(1-z)|{\tilde{\varvec{q}}}^2-(1-z)^2{\varvec{k}}^2|} + \frac{z^2{\tilde{\varvec{q}}}^2-z(1-z)(1-3z+z^2){\varvec{k}}^2}{(1-z)({\tilde{\varvec{q}}}^2+z(1-z){\varvec{k}}^2)} \right] .\nonumber \\ \end{aligned}$$

It is easy to check that the above splitting functions reduce to the collinear DGLAP results when ${\varvec{k}}^2\rightarrow 0$.

### The gluon-to-gluon splitting function

The real part of the ${\tilde{P}}_{gg}$ splitting function is given by the matrix element originating from the diagram in Fig. 1d

64 $$\begin{aligned} g^2\, \delta \left( (k-q)^2\right) \, W_{gg}= & {} \mathbb {P}_{g,\,\text {in}} \otimes \hat{K}_{gg}^{(0)}(q, k) \otimes \mathbb {P}_{g,\,\text {out}} \nonumber \\= & {} \mathbb {P}_{g,\,\text {in}}^{\beta \beta '}(k) \, \mathbb {P}_{g,\,\text {out}}^{\mu '\nu '}(q) (\Gamma _{g^*g^*g}^{\beta \mu \alpha })^\dagger \Gamma _{g^*g^*g}^{\nu \beta '\alpha '} \nonumber \\&\times \frac{-i d^{\mu \mu '}(q)}{q^2 - i\epsilon } \, \frac{id^{\nu \nu '}(q)}{q^2 + i \epsilon } \,d^{\alpha \alpha '}(k-q),\nonumber \\ \end{aligned}$$

where $\Gamma _{g^*g^*g}^{\mu \nu \alpha }$ is the effective gluon production vertex of Eq. (52) and we use the newly defined gluon projector Eq. (51). We obtain the following ${\tilde{P}}_{gg}$ splitting function

65 $$\begin{aligned} {\tilde{P}}_{gg}^{(0)} (z, {\tilde{\varvec{q}}}, {\varvec{k}})&= 2 C_A\, \left\{ \frac{{\tilde{\varvec{q}}}^4}{\left( {\tilde{\varvec{q}}}-(1-z){\varvec{k}}\right) ^2[{{\tilde{\varvec{q}}}^2+z(1-z){\varvec{k}}^2}]}\right. \nonumber \\&\quad \times \left. \left[ \frac{z}{1-z} + \frac{1-z}{z} + (3-4z) \frac{{\tilde{\varvec{q}}}\cdot {\varvec{k}}}{ {\tilde{\varvec{q}}}^2} + z(3-2z) \frac{{\varvec{k}}^2}{{\tilde{\varvec{q}}}^2} \right] \right. \nonumber \\&\quad \left. +\, \frac{(1 + \epsilon ){\tilde{\varvec{q}}}^2 z(1-z) [2 {\tilde{\varvec{q}}}\cdot {\varvec{k}}+ (2z -1) {\varvec{k}}^2]^2}{2 {\varvec{k}}^2 [{\tilde{\varvec{q}}}^2+z(1-z){\varvec{k}}^2]^2} \right\} . \end{aligned}$$

After angular averaging (and setting $\epsilon =0$) this provides

66 $$\begin{aligned}&\bar{P}_{gg}^{(0)}\left( z, \frac{{\varvec{k}}^2}{{\tilde{\varvec{q}}}^2} \right) \nonumber \\&\quad = C_A\, \frac{{\tilde{\varvec{q}}}^2}{{\tilde{\varvec{q}}}^2+z(1-z){\varvec{k}}^2} \left[ \frac{(2-z){\tilde{\varvec{q}}}^2+(z^3-4z^2+3z){\varvec{k}}^2}{z(1-z)\left| {\tilde{\varvec{q}}}^2-(1-z)^2{\varvec{k}}^2\right| }\right. \nonumber \\&\qquad \left. +\, \frac{(2z^3-4z^2+6z-3){\tilde{\varvec{q}}}^2+z(4z^4-12z^3+9z^2+z-2){\varvec{k}}^2}{(1-z)({\tilde{\varvec{q}}}^2+z(1-z){\varvec{k}}^2)} \right] .\nonumber \\ \end{aligned}$$

### Kinematic limits

As a next step we verify the necessary kinematic limits which the kernel needs to obey. In the collinear limit this is straightforward, since the transverse integral in Eq. (2) is specially adapted for this limit. In particular, one easily obtains the real part of the DGLAP gluon-to-gluon splitting function:^6^

67 $$\begin{aligned} \lim _{{\varvec{k}}^2 \rightarrow 0} \bar{P}_{ij}^{(0)}\left( z, \frac{{\varvec{k}}^2}{{\tilde{\varvec{q}}}^2}\right)&= 2\, C_A\, \left[ \frac{z}{1-z} + \frac{1-z}{z} + z\,\left( 1-z\right) \right] . \end{aligned}$$

In order to study the behaviour of the obtained splitting kernel in the high energy and soft limit, it is useful to change the variables of integrations in the TMD kernel Eq. (2) which will be particularly useful to disentangle $z\rightarrow 1$ and the ${{\tilde{\varvec{q}}}\rightarrow (1-z) {\varvec{k}}}$ singularities. With ${\tilde{\varvec{p}}}= \frac{{\varvec{k}}- { \varvec{q}}}{1-z} = {\varvec{k}}- \frac{{\tilde{\varvec{q}}}}{1-z}$ and changing variables accordingly we have

68 $$\begin{aligned}&{\hat{K}}_{gg} \left( z, \frac{{\varvec{k}}^2}{\mu ^2}, \epsilon , \alpha _s \right) \nonumber \\&\quad = C_A\, \frac{\alpha _s}{2\pi } \, z\, \frac{e^{-\epsilon \gamma _E}}{\mu ^{2\epsilon }} \int \frac{d^{2+2\epsilon }{\tilde{\varvec{p}}}}{ \pi ^{1+\epsilon }} \, \nonumber \\&\qquad \times \Theta \left( \mu _F^2 - ( (1-z) ({\varvec{k}}- {\tilde{\varvec{p}}})^2 + z {\varvec{k}}^2)\right) \, \nonumber \\&\qquad \times (1-z)^{2\epsilon } \left[ \frac{2}{(1-z)z\, {\tilde{\varvec{p}}}^2} + \frac{2 ({\tilde{\varvec{p}}}\cdot {\varvec{k}}- 2 (1-z) {\tilde{\varvec{p}}}^2 )}{{\tilde{\varvec{p}}}^2 [ (1-z) ({\varvec{k}}- {\tilde{\varvec{p}}})^2 + z {\varvec{k}}^2 ] }\right. \nonumber \\&\qquad \left. +\, (1+ \epsilon )\, \frac{z(1-z) }{ {\varvec{k}}^2 } \left( \frac{{\varvec{k}}^2 - 2 (1-z) {\tilde{\varvec{p}}}\cdot {\varvec{k}}}{ (1-z) ({\varvec{k}}- {\tilde{\varvec{p}}})^2 + z {\varvec{k}}^2} \right) ^2 \right] \end{aligned}$$

where

69 $$\begin{aligned} - q^2&= (1-z) ({\varvec{k}}- {\tilde{\varvec{p}}})^2 + z {\varvec{k}}^2\,, \end{aligned}$$

yields precisely the absolute value of the virtuality of the *t*-channel gluon. First we note that in the collinear limit ${\tilde{\varvec{p}}}^2 \gg {\varvec{k}}^2$ we re-obtain the DGLAP splitting function, Eq. (67), also in this parametrization. In the high energy limit $z \rightarrow 0$ we obtain

70 $$\begin{aligned} \lim _{z\rightarrow 0} {\hat{K}}_{gg} \left( z, \frac{{\varvec{k}}^2}{\mu ^2}, \epsilon , \alpha _s \right)&= \frac{\alpha _s C_A}{\pi (e^{\gamma _E}\mu ^2)^\epsilon }\int \frac{d^{2 + 2 \epsilon } {\tilde{\varvec{p}}}}{\pi ^{1 + \epsilon }} \nonumber \\&\quad \times \Theta \left( \mu _F^2 - ({\varvec{k}}- {\tilde{\varvec{p}}})^2\right) \frac{1}{{\tilde{\varvec{p}}}^2} \nonumber \\&= \int \frac{d^{2 + 2 \epsilon } { \varvec{q}}}{\pi ^{1 + \epsilon }} \Theta \left( \mu _F^2 -{{ \varvec{q}}}^2\right) \nonumber \\&\quad \times \frac{\alpha _s C_A}{\pi (e^{\gamma _E}\mu ^2)^\epsilon } \frac{1}{({ \varvec{q}}- {\varvec{k}})^2}, \end{aligned}$$

where the term under the integral is easily identified as the real part of the LO BFKL kernel. For the limit $z \rightarrow 1$, we find, in complete analogy,

71 $$\begin{aligned} \lim _{z\rightarrow 1} {\hat{K}}_{gg} \left( z, \frac{{\varvec{k}}^2}{\mu ^2}, \epsilon , \alpha _s \right)&= \frac{\alpha _s C_A}{\pi (e^{\gamma _E}\mu ^2)^\epsilon }\nonumber \\&\quad \times \int \frac{d^{2 + 2 \epsilon } {\tilde{\varvec{p}}}}{\pi ^{1 + \epsilon }} \Theta \left( \mu _F^2 - {\varvec{k}}^2\right) \frac{1}{{\tilde{\varvec{p}}}^2 (1-z)^{1-2\epsilon }} \nonumber \\&= \frac{\alpha _s C_A}{\pi (e^{\gamma _E}\mu ^2)^\epsilon }\int \frac{d^{2 + 2 \epsilon } {\tilde{\varvec{p}}}}{\pi ^{1 + \epsilon }} \Theta \left( \mu _F^2 - {\varvec{k}}^2\right) \nonumber \\&\quad \times \frac{1}{{\tilde{\varvec{p}}}^2} \left( \frac{1}{2 \epsilon } \delta (1-z) + \frac{1}{(1-z)_+^{1-2\epsilon }}\right) , \end{aligned}$$

where in the last term we made use of the plus prescription to isolate the singularity at $z=1$ (see [2] or any QCD standard reference for the precise definition).

Another limit of interest is the vanishing transverse momentum of the produced gluon ${\tilde{\varvec{p}}}\rightarrow 0$. If we parametrize the phase space of the produced gluon in terms of its transverse momentum, rapidity and azimuthal angle, this limit corresponds to the soft limit, *i.e.* the limit where the four momentum of the produced gluon vanishes. To isolate this singularity, we introduce a phase space slicing parameter $\lambda$, to separate the potential divergent region ($|{\tilde{\varvec{p}}}| < \lambda$) from the finite transverse momentum integral ($|{\tilde{\varvec{p}}}| > \lambda$). Taking the limit $\lambda \rightarrow 0$ we find for the divergent part of the TMD kernel,

72 $$\begin{aligned} {\hat{K}}_{gg}^{\text {div.}} \left( z, \frac{{\varvec{k}}^2}{\mu ^2}, \epsilon , \alpha _s \right)&=\Theta (\mu _F^{2} - {\varvec{k}}^2) \frac{\alpha _s C_A}{\pi } \frac{e^{-\gamma _E \epsilon }}{\epsilon \Gamma (1 + \epsilon )} \left( \frac{\lambda ^2}{\mu ^2} \right) ^\epsilon \nonumber \\&\quad \times \frac{1}{(1-z)^{1-2\epsilon } z} \nonumber \\&=\Theta (\mu _F^{2} - {\varvec{k}}^2) \frac{\alpha _s C_A}{\pi } \frac{e^{-\gamma _E \epsilon }}{\epsilon \Gamma (1 + \epsilon )} \left( \frac{\lambda ^2}{\mu ^2} \right) ^\epsilon \nonumber \\&\quad \times \left[ \frac{1}{2\epsilon } \delta (1-z) + \frac{1}{(1-z)_+^{1-2\epsilon } z} \right] . \end{aligned}$$

As usually the ${\tilde{\varvec{p}}}\rightarrow 0$ and the $z\rightarrow 1$ singularity are regularized by dimensional regularization, while the high energy singularity requires a separate regulator (or needs to be controlled by energy conservation). Apart from the above extraction of singularities, it is also interesting to simply consider the ${\tilde{\varvec{p}}}\rightarrow 0$ limit of the TMD kernel, where set for the time being $\epsilon \rightarrow 0$; one obtains

73 $$\begin{aligned} {\hat{K}}_{gg} \left( z, \frac{{\varvec{k}}^2}{\mu ^2}, 0, \alpha _s \right)&= z\, \int _{{\tilde{\varvec{p}}}^2_{min}}^{{\tilde{\varvec{p}}}^2_{max}} \frac{d {\tilde{\varvec{p}}}^2}{{\tilde{\varvec{p}}}^2} \frac{\alpha _s C_a}{\pi }\left[ \frac{1}{z} + \frac{1}{1-z} + \mathcal {O}\left( \frac{{\tilde{\varvec{p}}}^2}{{\varvec{k}}^2}\right) \right] , \end{aligned}$$

which allows to identify the terms under the integrand as the (real part/unresummed^7^) CCFM-kernel. Note that we did not take any dedicated measures to arrive at this limit; it merely appears as a natural by-product of the conditions imposed on the TMD splitting kernel.

## Summary and discussion

The main result of this paper is the calculation of a transverse momentum dependent gluon-to-gluon splitting function. The splitting function reduces both to the conventional gluon-to-gluon DGLAP splitting function in the collinear limit as well as to the LO BFKL kernel in the low *x*/high energy limit; moreover the CCFM gluon-to-gluon splitting function is re-obtained in the limit where the transverse momentum of the emitted gluon vanishes, *i.e.* if the emitted gluon is soft. The derivation of this result is based on the Curci-Furmanski-Petronzio formalism for the calculation of DGLAP splitting functions in axial gauges. To address gauge invariance in the presence of off-shell partons, high energy factorization adapted for axial gauges has been used to derive an effective production vertex which then could be shown to satisfy current conservation.

The next step in completing the calculation of TMD splitting functions is the determination of the still missing virtual corrections, which will be carried out using the techniques developed in [51–56] within Lipatov’s high energy effective action and/or the helicity spinor framework [57]. With the complete set of splitting functions at hand, it will be finally possible to formulate an evolution equation for the unintegrated (TMD) parton distribution functions including both gluons and quarks. As a final goal, we have in mind to use this evolution equation to formulate a parton shower algorithm, which can be implemented into a new Monte Carlo program with TMD splitting functions, extending currently available codes such as Cascade [58]. This will allow to address, with new theoretical tools within the TMD approach, both low *x* and especially moderate *x* phenomenology [59].

Another direction for future research will aim at clarifying the relation of our result with the linearised results for TMD evolution in the low and intermediate *x* region obtained in [60]. While both results agree in the collinear and the high energy limit, they differ for more general kinematics. We believe that the origin of this difference lies in an observation made in [61], see also [62], namely the existence of 2 different gluon distributions in the low *x* limit. While the TMD distribution studied in [60] – which agrees in the intermediate *x* region with the definition provided in [63] – seems to correspond to the Weizsäcker-Williams gluon distribution (in the terminology of [61]), the gluon distribution obtained from “unintegrating” the collinear gluon distribution underlying the CFP-formalism, appears at least at first sight related to what [61] call the Fourier-transform of the dipole distribution; in particular it is said dipole distribution which enters inclusive observables in the high energy limit *e.g.* proton structure functions. A detailed answer and study of this question is beyond the scope of this paper and will be addressed in a separate publication. In particular, such a research also needs to answer the question as to which extent the obtained TMD kernels can be derived independently from the use of the Curci–Furmanski–Petronzio formalism, which is constrained to the use of axial light-cone gauges.

## A Conventions for the spinor helicity amplitudes

We repeat here the parametrisation of the momenta used in [36, 37], which reads

74 $$\begin{aligned}&k_1^\mu + k_2^\mu + \cdots + k_n^\mu = 0 \quad \text {momentum conservation} \end{aligned}$$

75 $$\begin{aligned}&p_1^2 = p_2^2 = \cdots = p_n^2 = 0 \quad \text {light-likeness} \end{aligned}$$

76 $$\begin{aligned}&\,p_1\!\cdot \!k_1\, = \,p_2\!\cdot \!k_2\,=\cdots =\,p_n\!\cdot \!k_n\,=0 \quad \text {eikonal condition} \end{aligned}$$

where for every $k^\mu _i$ there is a corresponding orthogonal, light-like direction $p^\mu _i$.

With the help of an auxiliary light-like four-vector $q^\mu$, the momentum $k^\mu$ can be decomposed in terms of its light-like direction $p^\mu$, satisfying $\,p\!\cdot \!k\,=0$, and a transversal part, following

77 $$\begin{aligned} k^\mu = x(q)p^\mu - \frac{\kappa }{2}\,\frac{\langle p|\gamma ^\mu |q]}{[pq]} - \frac{\kappa ^*}{2}\,\frac{\langle q|\gamma ^\mu |p]}{\langle qp\rangle }, \end{aligned}$$

with

78 $$\begin{aligned} x(q)=\frac{\,q\!\cdot \!k\,}{\,q\!\cdot \!p\,},\quad \kappa = \frac{\langle q|K \! \! \! /|p]}{\langle qp\rangle },\quad \kappa ^*= \frac{\langle p|K \! \! \! /|q]}{[pq]}. \end{aligned}$$

If the momentum *k* is on-shell, then it coincides with its associated direction, $p=k$.

79 $$\begin{aligned} k^2 = -\kappa \kappa ^*\ . \end{aligned}$$

We focus on the fundamental *colour-ordered* or *dual* amplitudes, where the gauge-group factors have been stripped off and all particles are massless. By construction, these amplitudes contain only planar Feynman graphs and are constructed with colour-stripped Feynman rules.

The polarization vectors for gluons can be expressed as

80 $$\begin{aligned} \epsilon ^\mu _{+ } = \frac{\langle q | \gamma ^\mu | g]}{\sqrt{2}\langle q g\rangle }, \quad \epsilon ^\mu _{-} = \frac{\langle g |\gamma ^\mu | q]}{\sqrt{2}[g q]}, \end{aligned}$$

where *q* is the auxiliary light-like vector and *g* stands for the gluon momentum. In the expressions of the amplitudes, gluons will be denoted by the number of the corresponding particle, whereas we will always explicitly distinguish quarks and antiquarks with *q* and $\bar{q}$ respectively.

Finally, the polarization vectors for the auxiliary photons coming in pairs with the off-shell quarks are

81 $$\begin{aligned} \epsilon ^\mu _{f\,+} = \frac{\langle q | \gamma ^\mu | f]}{\sqrt{2}\langle q f\rangle }, \quad \epsilon ^\mu _{f\,-} = \frac{\langle f |\gamma ^\mu | q]}{\sqrt{2}[f q]}, \end{aligned}$$

where *f* denotes the fermion momentum spinor and *q* is again the auxiliary vector.

Further information on the spinor helicity formalism can be found in [36–38].

## B Real emission splitting functions in a set of variables more suitable for an evolution equation

In this section we provide the TMD splitting function in terms of a different set of variables, that was used in Ref. [3] in the construction of an evolution equation. Namely, we will use the real emitted momentum ${ \varvec{p}}'={\varvec{k}}-{ \varvec{q}}$ and the outgoing momentum ${ \varvec{q}}$, see Fig. 1.

The definition of the kernel corresponding to Eq. (8) in these variables reads

82 $$\begin{aligned} {\hat{K}}_{ij} \left( z, \frac{{\varvec{k}}^2}{\mu ^2}, \epsilon , \alpha _s \right) \!\!= & {} \! \frac{\alpha _s \cdot z}{2\pi \mu ^{2\epsilon } e^{\epsilon \gamma _E}} \int \!\frac{d^{2+2\epsilon }{ \varvec{p}}'}{\pi ^{1+\epsilon } { \varvec{p}}'^2}\nonumber \\&\times {\tilde{P}}_{ij}^{(0)}(z,{ \varvec{q}},{ \varvec{p}}') \Theta \left( \mu _F^2\! -\! \frac{z{ \varvec{p}}'^2 }{1\!-\!z}\! -\! { \varvec{q}}^2\right) .\nonumber \\ \end{aligned}$$

In the following we list the splitting functions in the new variables.

### B.1 Angular and transverse momentum dependent TMD splitting functions

83 $$\begin{aligned} {\tilde{P}}_{qg}^{(0)} (z,{ \varvec{q}},{ \varvec{p}}')= & {} T_R\, \frac{{ \varvec{p}}'^2}{({ \varvec{p}}'+{ \varvec{q}})^2}\nonumber \\&\times \,\bigg [ 1 + \frac{2({ \varvec{p}}'\cdot { \varvec{q}})}{z{ \varvec{p}}'^2+(1-z){ \varvec{q}}^2} + \frac{{ \varvec{p}}'^2{ \varvec{q}}^2}{(z{ \varvec{p}}'^2+(1-z){ \varvec{q}}^2)^2} \bigg ], \end{aligned}$$

84 $$\begin{aligned} {\tilde{P}}_{gq}^{(0)} (z,{ \varvec{q}},{ \varvec{p}}')= & {} C_F\, \frac{z{ \varvec{p}}'^4 + 2\left( z+\frac{1}{z}-2\right) { \varvec{q}}^4 + 2(1-z){ \varvec{p}}'^2{ \varvec{q}}^2}{(z{ \varvec{p}}'^2+(1-z){ \varvec{q}}^2)^2} \nonumber \\&+\, \epsilon \, C_F \frac{z{ \varvec{p}}'^4}{(z{ \varvec{p}}'^2+(1-z){ \varvec{q}}^2)^2}, \end{aligned}$$

85 $$\begin{aligned} {\tilde{P}}_{qq}^{(0)} (z,{ \varvec{q}},{ \varvec{p}}')= & {} C_F\, \left[ \frac{2}{1-z} + \frac{2({ \varvec{p}}'\cdot { \varvec{q}})}{z{ \varvec{p}}'^2+(1-z){ \varvec{q}}^2} + \frac{(1-z){ \varvec{p}}'^2{ \varvec{q}}^2}{(z{ \varvec{p}}'^2+(1-z){ \varvec{q}}^2)^2} \right] \nonumber \\&+ \,\epsilon \, C_F \frac{(1-z){ \varvec{p}}'^2{ \varvec{q}}^2}{(z{ \varvec{p}}'^2+(1-z){ \varvec{q}}^2)^2}, \end{aligned}$$

86 $$\begin{aligned} {\tilde{P}}_{gg}^{(0)} (z,{ \varvec{q}},{ \varvec{p}}')= & {} C_A\, \left[ \frac{{ \varvec{p}}'^4{ \varvec{q}}^2}{({ \varvec{p}}'+{ \varvec{q}})^2(z{ \varvec{p}}'^2+(1-z){ \varvec{q}}^2)^2}\right. \nonumber \\&\left. - \frac{(2z-1){ \varvec{p}}'^2{ \varvec{q}}^2 + 2z{ \varvec{q}}^2({ \varvec{p}}'\cdot { \varvec{q}}) + 4({ \varvec{p}}'\cdot { \varvec{q}})^2}{({ \varvec{p}}'+{ \varvec{q}})^2(z{ \varvec{p}}'^2+(1-z){ \varvec{q}}^2)}\right. \nonumber \\&\left. - \,\frac{z^2(1+z^2){ \varvec{p}}'^4 + z(1-z)(3+z^2){ \varvec{p}}'^2{ \varvec{q}}^2 + 2(1-z)^2{ \varvec{q}}^4}{z(1-z)(z{ \varvec{p}}'^2+(1-z){ \varvec{q}}^2)^2}\right. \nonumber \\&\left. -\, \frac{2({ \varvec{p}}'\cdot { \varvec{q}})}{({ \varvec{p}}'+{ \varvec{q}})^2} \right] - \epsilon \, C_A \frac{z(1-z){ \varvec{p}}'^2({ \varvec{p}}'^2-{ \varvec{q}}^2)^2}{({ \varvec{p}}'+{ \varvec{q}})^2(z{ \varvec{p}}'^2+(1-z){ \varvec{q}}^2)^2}. \end{aligned}$$

### B.2 Angular averaged TMD splitting functions

The angular averaged splitting functions listed below are for $\epsilon =0$.

87 $$\begin{aligned} \bar{P}_{qg}^{(0)}= & {} T_R\, \left[ \frac{{ \varvec{p}}'^2}{z{ \varvec{p}}'^2+(1-z){ \varvec{q}}^2} - \frac{z(1-z){ \varvec{p}}'^2\left| { \varvec{p}}'^2-{ \varvec{q}}^2\right| }{\left( z{ \varvec{p}}'^2+(1-z){ \varvec{q}}^2\right) ^2} \right] , \end{aligned}$$

88 $$\begin{aligned} \bar{P}_{gq}^{(0)}= & {} C_F\, \frac{z{ \varvec{p}}'^4 + 2(1-z){ \varvec{p}}'^2{ \varvec{q}}^2 + 2\left( z+\frac{1}{z}-2\right) { \varvec{q}}^4}{\left( z{ \varvec{p}}'^2+(1-z){ \varvec{q}}^2\right) ^2}, \end{aligned}$$

89 $$\begin{aligned} \bar{P}_{qq}^{(0)}= & {} C_F\, \left[ \frac{2}{1-z} + \frac{(1-z){ \varvec{p}}'^2{ \varvec{q}}^2}{\left( z{ \varvec{p}}'^2+(1-z){ \varvec{q}}^2\right) ^2} \right] , \end{aligned}$$

90 $$\begin{aligned} \bar{P}_{gg}^{(0)}= & {} C_A\, \left[ - \frac{2}{z(1-z)} + \frac{2{ \varvec{p}}'^2}{z{ \varvec{p}}'^2+(1-z){ \varvec{q}}^2} + \frac{{ \varvec{p}}'^2}{\left| { \varvec{p}}'^2-{ \varvec{q}}^2\right| }\right. \nonumber \\&-\, \frac{{ \varvec{p}}'^4+{ \varvec{p}}'^2{ \varvec{q}}^2}{\left| { \varvec{p}}'^2-{ \varvec{q}}^2\right| \left( z{ \varvec{p}}'^2+(1-z){ \varvec{q}}^2\right) }\nonumber \\&\left. +\, \frac{{ \varvec{p}}'^4{ \varvec{q}}^2}{\left| { \varvec{p}}'^2-{ \varvec{q}}^2\right| \left( z{ \varvec{p}}'^2+(1-z){ \varvec{q}}^2\right) ^2} \right] . \end{aligned}$$

## Footnotes

1 For a review see [8].

2 As stressed in [35], these auxiliary ‘photons’ interact only with the corresponding auxiliary fermion and not with any other fermion with electromagnetic charge; they can actually be thought of as bosons mediating an extra fictitious abelian interaction. Since the purpose of their introduction is to build an amplitude which is off-shell and gauge-invariant, and whose dependence from the auxiliary particles is eventually dropped, this is legitimate.

3 The proliferation of $\sqrt{2}$ factors is typical for the colour-ordered Feynman rules [36, 39].

4 See [44] for a formulation of the corresponding program which does not require light-cone gauges.

5 This clearly has to be the case, but since the projector selects only the infrared poles, which come anyway from the region of low $l^2$, the upper limit of the integral is actually irrelevant. However, starting from NLO more complicated forms of the upper phase space limit can lead to the dependence of the splitting functions on this choice [46].

6 We verified this limit also for finite $\epsilon$ where it holds equally.

7 Both Sudakov and Non-Sudakov-form factors are obviously absent and the lower and upper limits of integration are result of adding virtual corrections and appropriate angle related scale of hard process.
